# Efficacy of *Bacillus thuringiensis* var. *israelensis* against malaria mosquitoes in northwestern Burkina Faso

**DOI:** 10.1186/1756-3305-7-371

**Published:** 2014-08-15

**Authors:** Peter Dambach, Valérie R Louis, Achim Kaiser, Saidou Ouedraogo, Ali Sié, Rainer Sauerborn, Norbert Becker

**Affiliations:** Institute of Public Health, University of Heidelberg, Heidelberg, Germany; German Mosquito Control Association (KABS), Speyer, Germany; Centre de Recherche en Santé de Nouna (CRSN), Nouna, Burkina Faso; Centre for Organismal Studies, University of Heidelberg, Heidelberg, Germany

**Keywords:** *Anopheles*, Malaria, *Bacillus thuringiensis israelensis*, Mosquito control, Water dispersible granule, Burkina Faso, West Africa

## Abstract

**Background:**

In Sub Saharan Africa malaria remains one of the major health problems and its control represents an important public health measure. Integrated malaria control comprises the use of impregnated mosquito nets and indoor residual spraying. The use of drugs to treat patients can create additional pressure on the equation of malaria transmission. Vector control may target the adult mosquitoes or their aquatic larval stages. Biological larvicides such as *Bacillus thuringiensis israelensis* (*Bti*) represent a promising approach to support malaria control programs by creating additional pressure on the equation of malaria transmission.

**Methods:**

In this study we examined the efficacy of a water-dispersible granule formulation (WDG) of the biological larvicide *Bti* (VectoBac®) against wild *Anopheles* spp. larvae. Different concentrations of the larvicide were tested in standardized plastic tubs in the field against untreated controls. In weekly intervals tubs were treated with fixed concentrations of larvicide and the percentage reduction of larvae and pupae was calculated.

**Results:**

All used concentrations successfully killed 100 percent of the larvae within 24 hours, while the higher concentrations showed a slightly prolonged residual effect. Natural reconolization of larvae took place after two and three days respectively, late instar larvae were not found before 5 days after treatment. For the higher concentrations, up to three days no new larvae were found, implicating that the residual effect of WDG in tropical conditions is approximately one to two days. The overall pupae reduction in treated tubs was 98.5%.

**Conclusions:**

Biological larviciding with *Bti* can be a promising, additional tool in the fight against malaria in Africa. Environmental particularities in tropical Africa, first and foremost the rapid development of mosquitoes from oviposition to imago have to be taken into account before implementing such counter measures in national or international vector control programs. Nonetheless biological larviciding seems to be an appropriate measure for selected conditions, offering a significant contribution to the future of malaria control.

## Background

Some 40 years after the use of DDT was widely restricted as a universal tool for fighting against the vectors of numerous diseases, first and foremost malaria, larviciding has become an important component in the control of a multitude of vector borne infectious diseases. In its current deployment, however, the types of larvicides used, shifted towards ecologically sound toxins based on *Bacillus thuringiensis israelensis* (*Bti*) and *Bacillus sphaericus* (*Bs*), which selectively kill Culicidae larvae and cause no harm for flora and fauna. Being in place for decades in North America, Europe and parts of Asia, larval source management (LSM) based on biological larvicides has rarely left the frame of experimental application in countries of Sub Saharan Africa. Only recently the WHO recommended LSM as a supplemental measure, in some environmental settings, to the package of integrated malaria control measures [1].

In contrast to most chemical larvicides *Bti* has a low resistance potential and virtually no losses in efficacy have been observed in the field following operational use [2–4]. Resistance to *Bs* has been reported [5, 6] but seems to play a minor part in field application, in particular when *Bs* is combined with *Bti*.

Depending on the environmental setting, and in particular, the number, size and accessibility of breeding sites, larviciding with *Bti* can be an important additional tool for vector population control in integrated control programs. In many environmental settings larvae are found in high densities in their respective breeding sites and can be easily accessed, therefore, adult vector populations can be reduced by orders of magnitude with LSM [7–9]. In tropical Africa though, those larvicides have certain limitations and need careful testing under field conditions before being used in vector control. Higher temperatures increase larvicide efficacy, partially due to an increased larval feeding rate [10, 11] but may foster biodegradation [12]. Dilution by heavy rainfall, wind drift of surface water and interacting flora and fauna may influence the efficacy and residual activity. In addition the thermal conditions lead to a drastically shortened reproduction cycle of vector mosquitoes, heavily influencing the need for retreatment of breeding sites. Each geographic region features a characteristic combination of those factors and gives reason for testing the larvicide’s efficacy under the respective field conditions. It is the first time of testing *Bti* in that region, which was performed within the framework of a large scale intervention program which uses satellite derived risk maps to predict larval densities [13].

### Objective

To evaluate the effect of larviciding with different concentrations of a *Bacillus thuringiensis israelensis* strain AM65-52 formulation (Vectobac®, WDG) on larval populations of wild malaria vectors *Anopheles* spp., its residual effect and the natural reconolization in semi-field conditions.

## Methods

### Study area

All experiments were performed at the CRSN (Centre de Recherche en Santé de Nouna) research center in Nouna, Northwestern Burkina Faso. The region is characterized as dry orchard savannah with sub-Saharan climate, and an annual mean precipitation of about 800 mm and a mean temperature of 27.8°C. The altitude at which the research center is situated, as well as the surrounding is between 250 and 300 m above sea-level. The area around the small town of Nouna is rural, with most people living from subsistence farming. Malaria incidence in the region is extremely high and shows endemicity with a marked peak during the late rainy season. Despite the strong seasonality, malaria transmission is sustained all year round. The Entomologic Inoculation Rate (EIR), representing the number of infective bites per person per year is around 700. The region shows a rainy season, usually between June and September and a dry season from November to April with phases of transition in between. Whilst during the rainy season large water accumulations such as rice fields, ponds, brickworks etc. in and around villages act as mosquito breeding sites, the dry season shows a completely different picture. With ceasing rains only few ponds and riverbeds continue bearing water, vegetation is sparse and day temperatures regularly rise above 40°C. Main vectors for malaria are *Anopheles gambiae sl*. with more than 98% and to a much smaller extent *A. funestus* and *A. nili*.

### Open field trials

Two rounds of open field trials were performed between October and December 2012, corresponding to the late rainy season (round 1) and beginning of the dry season (round 2). Twenty plastic tubs with a diameter of 60 cm were buried into an open field in an array of five rows with 4 tubs each, following the experimental design of Fillinger *et al.*[4]. Distance between rows was one meter, and between tubs in a row 2 meters. Mud soil and small amounts of typical vegetation from known *Anopheles* breeding sites were put into each tub, creating a standardized environment with suitable breeding conditions. Tubs were subsequently filled up to initial level with water from a nearby well. Each tub received 25 liters of water, representing an average water depth of 25 centimeters.

Prior to the initial treatment round, 100 field collected Anopheles spp. larvae consisting of 50 first and second instar and 50 third and fourth instar were placed in each tub. Prior to the second treatment round, 70 larvae were placed in each tub, consisting of 35 first and second instar and 35 third and fourth instar. *Anopheles* spp. larvae were collected from surrounding natural habitats for which earlier studies showed to be more than 95% *Anopheles gambiae s.l.*.

*Bti* strain AM65-52 formulation, VectoBac® WDG from Valent BioSciences Corp, Illinois, USA with a potency of 3000 ITUs/mg (International Toxic Units), lot number 215-413-PG was used for the treatments. Concentrations were calculated for a standard water depth of 10 cm, given a constant surface area [14]. Concentrations were 0.2; 0.4; 0.8 and 1.0 mg/l, which equals a surface application of 0.2-1.0 kg/ha. Four tubs in a row received the same concentration or served as control respectively. Initial *Bti* concentrations were fixed on the basis of earlier studies [4].

On days 0, 7, 14 and 21, 150 ml of WDG formulations in the respective concentration were applied evenly over the water surface to each tub, using a handheld dispenser. During each sampling round of 28 days a daily exhaustive larval count was performed in all 20 tubs, pupae were removed. All larvae were determined to genus level and *Anopheles* larvae additionally discriminated by larval stage (first and second instar hereafter called early instars, third and fourth instar hereafter called late instars). The percentage reduction in larval numbers was calculated using the formula introduced by Mulla [15], taking into account a natural alteration of mosquito larvae by biotic and abiotic factors in both, the treated and the control sites.


C1 and C2 describe the pre and post treatment densities of mosquito larvae in the control group and T1 and T2 in the pre and post treatment average numbers in the tubs with larvicide application. The values of C1 and T1 refer to the initial average numbers of larvae and change on the day of larvicide application. Recoveries of larval populations are shown as a reduction of zero percent, although Mulla’s formula would give negative values in this case. This attributes best to describe the efficacy of *Bti*, which has no effect for values below zero. For the test of statistical significance in larval reduction after *Bti* treatment, the average number of early instars, late instars, and pupae in the control and treatment tubs were compared daily by non-parametric Kruskal–Wallis one-way anova on ranks (α = 0.05 and 0.1) using SAS 9.2., SAS Institute Inc. Cary. NC USA.

## Results

**Figure 1:**
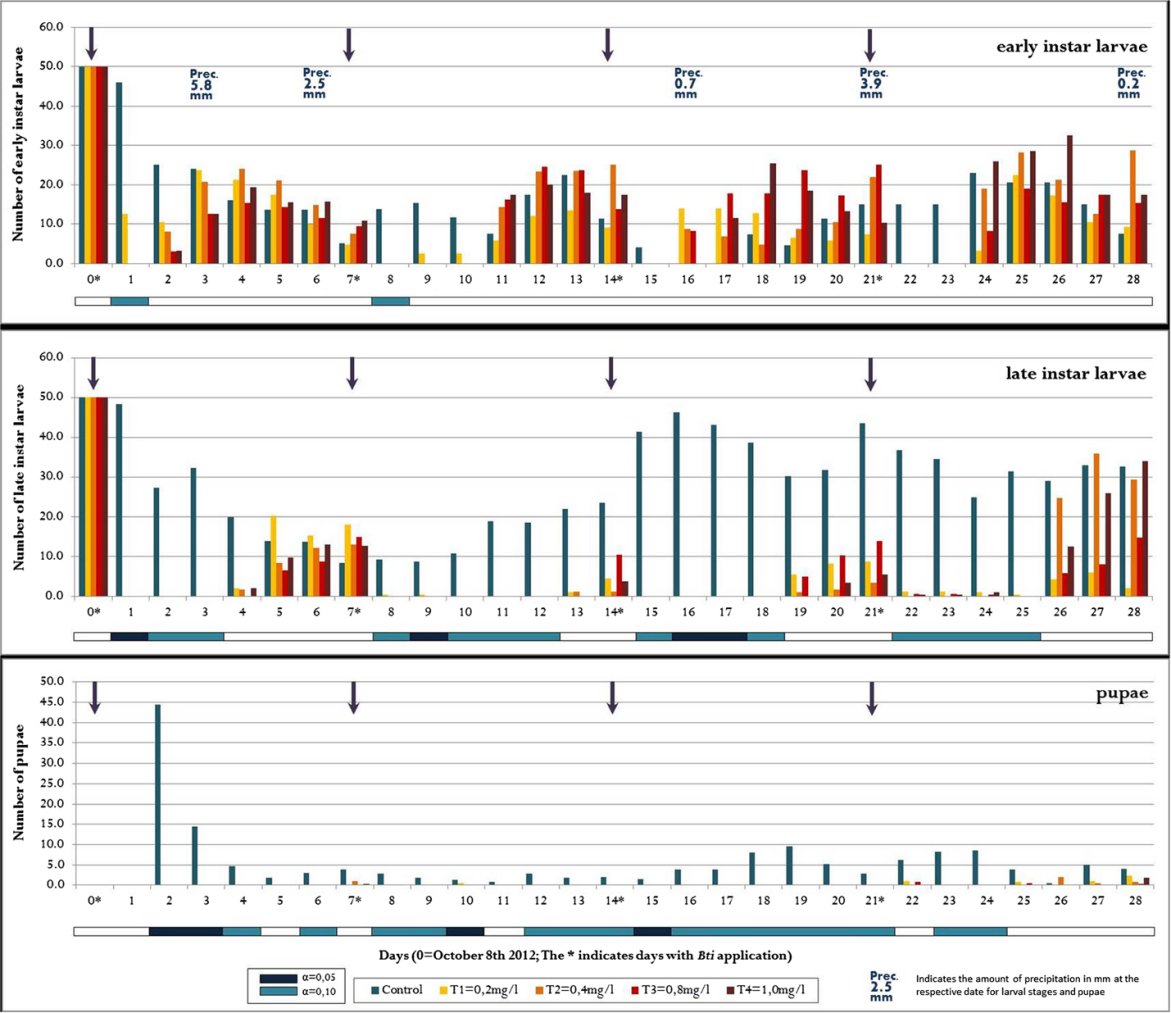
**Population dynamics of early larval instars, late instars and pupae of** ***Anopheles spp*** **. in open field trials exposed to water-dispersible granules (WDG) of** ***Bti*** **.** Arrows indicate the date of treatment. Trial period from October 8th to November 5th 2012. White horizontal bars indicate no significant difference between treatment and control tubs, blue bars do (dark blue: α = 0,05; light blue α = 0,10).

**Figure 2:**
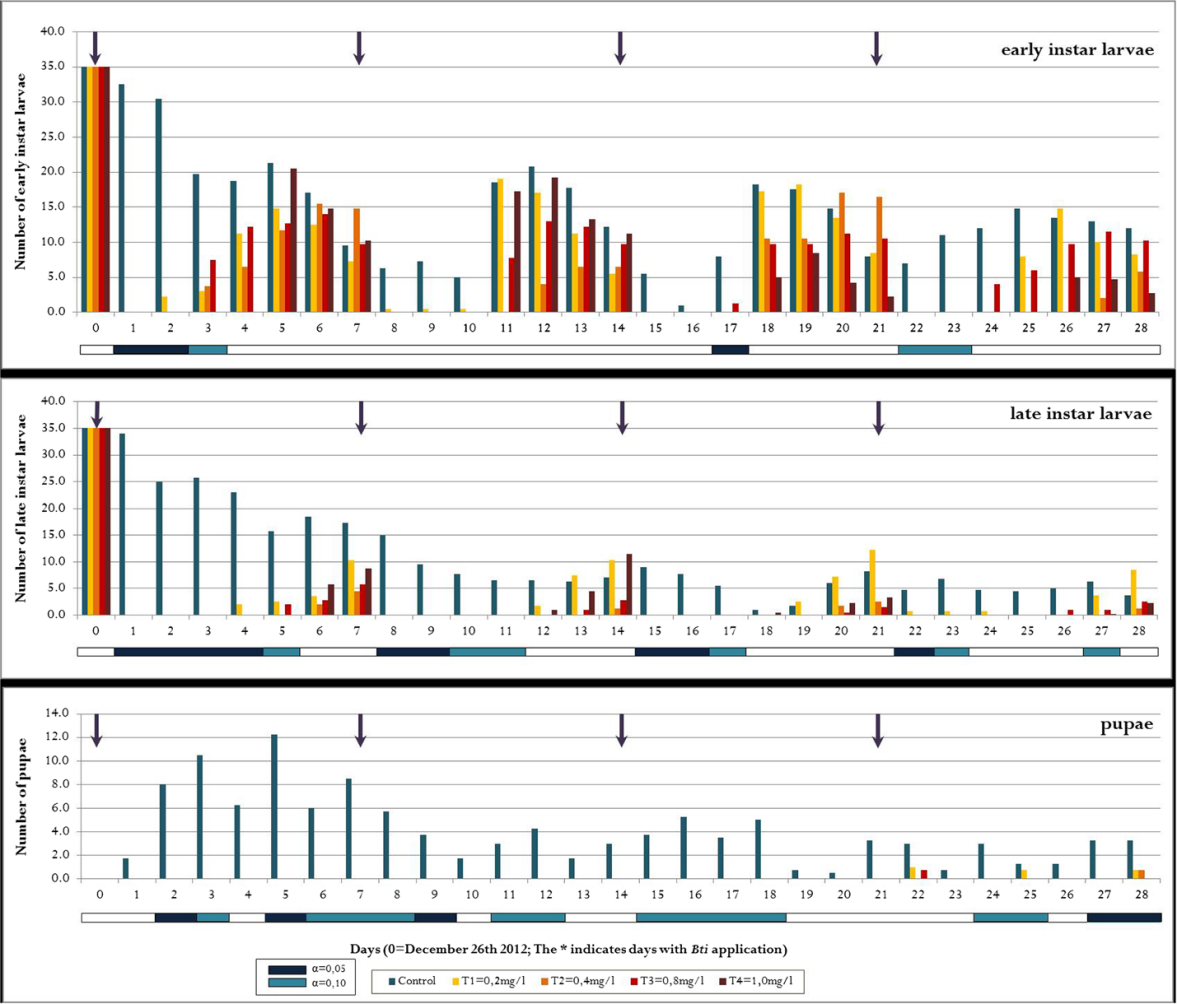
**Population dynamics of early larval instars, late instars and pupae of** ***Anopheles spp*** **. in open field trials exposed to water-dispersible granule (WDG) of** ***Bti*** **.** Arrows indicate the date of treatment. Trial period from December 26th 2012 to January 23rd 2013. White horizontal bars indicate no significant difference between treatment and control tubs, blue bars do (dark blue: α = 0,05; light blue α = 0,10). Days with precipitations are indicated and amount of rainfall is given in millimeters.

All concentrations tested showed a 100% reduction of larvae within the first 24 hours after application. Over time in both rounds (October/November 2012 and December/January 2012/13) and all sample cycles, natural declines and increases of larval densities were observed in the control and treatment groups. The first round of larvicide application showed reoccurrence of early instars after one to two days. In round two, early instar larvae were found after two days in the lowest concentration of 0.2 mg/l, and after three days in the higher concentrations. After five to six days in rounds one and two respectively, all concentrations showed late instar larvae. Pupae were only found in a small number of cases, mostly towards the end of each assay. During the first 21 days of each round virtually no pupae were found in the treated tubs. Despite the low residual effect of all WDG *Bti* concentrations of approximately two days and the fast recolonization with *Anopheles* larvae, the absence of pupae was significant. The overall pupal reduction in treated tubs averaged over both rounds was 98.5%, which is a proxy for the efficacy in reducing the emergence of adult mosquitoes. The second round of open field trials (from December 26th 2012 on) showed generally higher reduction rates and slightly longer residual effects. The lowest concentration showed in both rounds a shortened residual effect, which can be observed particularly amongst early instar larvae in the first and second application run. The detailed results are shown in Figure 1, Figure 2, and Table 1, Table 2.

**Table 1:** **Average number of Anopheles larvae and percentage reduction for different** ***Bti*** **concentrations (VectoBac® WDG) after larvicide application in open field trials from October 08th 2012 on**

	Average number per tub															Percentage reduction							
	Early instars					Late instars					Pupae					Early instars				Late instars			
Day	C	T1	T2	T3	T4	C	T1	T2	T3	T4	C	T1	T2	T3	T4	T1	T2	T3	T4	T1	T2	T3	T4
0*	50.0	50.0	50.0	50.0	50.0	50.0	50.0	50.0	50.0	50.0	0.0	0.0	0.0	0.0	0.0								
1	46.0	12.5	0.0	0.0	0.0	48.3	0.0	0.0	0.0	0.0	0.0	0.0	0.0	0.0	0.0	73	100	100	100	100	100	100	100
2	25.0	10.5	8.0	3.0	3.3	27.3	0.0	0.0	0.0	0.0	44.5	0.0	0.0	0.0	0.0	58	68	88	87	100	100	100	100
3	24.0	23.8	20.8	12.5	12.5	32.3	0.0	0.0	0.0	0.0	14.5	0.0	0.0	0.0	0.0	1	14	48	48	100	100	100	100
4	16.0	21.3	24.0	15.3	19.23	20.0	2.0	1.8	0.0	2.0	4.8	0.0	0.0	0.0	0.0	0	0	5	0	90	91	100	90
5	13.7	17.5	21.0	14.3	15.5	14.0	20.3	8.5	6.5	9.8	1.8	0.0	0.0	0.0	0.0	0	0	0	0	0	39	54	30
6	13.7	10.0	14.8	11.5	15.8	13.7	15.3	12.3	8.8	13.0	3.0	0.0	0.0	0.0	0.0	27	0	16	0	0	10	36	5
7*	5.0	4.8	7.5	9.5	10.8	8.3	18.0	13.0	15.0	12.8	3.8	0.0	1.0	0.0	0.3	5	0	0	0	0	0	0	0
8	13.8	0.0	0.0	0.0	0.0	9.3	0.5	0.0	0.0	0.0	2.8	0.0	0.0	0.0	0.0	100	100	100	100	97	100	100	100
9	15.3	2.5	0.0	0.0	0.0	8.8	0.5	0.0	0.0	0.0	1.8	0.0	0.0	0.0	0.0	83	100	100	100	97	100	100	100
10	11.8	2.5	0.0	0.0	0.0	10.8	0.0	0.0	0.0	0.0	1.3	0.5	0.0	0.0	0.0	78	100	100	100	100	100	100	100
11	7.5	5.8	14.3	16.3	17.5	19.0	0.0	0.0	0.0	0.0	0.8	0.0	0.0	0.0	0.0	19	0	0	0	100	100	100	100
12	17.5	12.0	23.3	24.5	20.0	18.5	0.0	0.0	0.0	0.0	2.8	0.0	0.0	0.0	0.0	28	11	26	47	100	100	100	100
13	22.5	13.5	23.5	23.8	18.0	22.0	1.0	1.3	0.0	0.0	1.8	0.0	0.0	0.0	0.0	37	30	44	63	98	96	100	100
14*	11.3	9.0	25.0	13.8	17.5	23.5	4.5	1.3	10.5	3.8	2.0	0.0	0.0	0.0	0.0	0	0	0	0	90	96	73	89
15	4.0	0.0	0.0	0.0	0.0	41.5	0.0	0.0	0.0	0.0	1.5	0.0	0.0	0.0	0.0	100	100	100	100	100	100	100	100
16	0.0	14.0	8.8	8.3	0.0	46.3	0.0	0.0	0.0	0.0	3.8	0.0	0.0	0.0	0.0	0	0	0	0	100	100	100	100
17	0.0	14.0	6.8	17.8	11.5	43.3	0.0	0.0	0.0	0.0	3.8	0.0	0.0	0.0	0.0	0	0	0	0	100	100	100	100
18	7.3	12.8	4.8	17.8	25.5	38.8	0.0	0.0	0.0	0.0	8.0	0.0	0.0	0.0	0.0	0	71	0	0	100	100	100	100
19	4.5	6.5	8.8	23.8	18.5	30.3	5.5	1.0	5.0	0.0	9.5	0.0	0.0	0.0	0.0	0	13	0	0	5	38	63	100
20	11.3	5.8	10.5	17.3	13.3	31.8	8.3	1.8	10.3	3.5	5.3	0.0	0.0	0.0	0.0	36	58	0	24	0	0	28	31
21*	15.0	7.3	22.0	25.0	10.3	43.5	8.8	3.5	14.0	5.5	2.8	0.0	0.0	0.0	0.0	40	34	0	56	0	0	28	21
22	15.0	0.0	0.0	0.0	0.0	36.8	1.3	0.0	0.8	0.5	6.3	1.0	0.0	0.8	0.0	100	100	100	100	82	100	94	89
23	15.0	0.0	0.0	0.0	0.0	34.5	1.3	0.0	0.8	0.5	8.3	0.0	0.0	0.0	0.0	100	100	100	100	82	100	93	89
24	23.0	3.3	19.0	8.3	26.0	25.0	1.0	0.0	0.5	1.0	8.5	0.0	0.0	0.0	0.0	71	44	78	0	80	100	94	68
25	20.5	22.5	28.3	19.0	28.5	31.5	0.5	0.0	0.0	0.0	3.8	0.8	0.0	0.5	0.0	0	6	44	0	92	100	100	100
26	20.5	17.3	21.3	15.5	32.5	29.0	4.3	24.8	5.8	12.5	0.5	0.0	2.0	0.0	0.0	0	29	55	0	27	0	38	0
27	15.0	10.5	12.5	17.5	17.5	33.0	6.0	36.0	8.0	26.0	5.0	1.0	0.5	0.0	0.0	0	43	30	0	10	0	25	0
28	7.5	9.3	28.8	15.3	17.5	32.8	2.0	29.5	14.8	34.0	4.0	2.3	0.8	0.3	1.8	0	0	0	0	70	0	0	0

*Asterisks indicate days with larvicide application. C = control, T_1_ = 0.2 mg/l, T_2_ = 0.4 mg/l, T_3_ = 0.8 mg/l, T_4_ = 1.0 mg/l.

**Table 2:** **Average number of Anopheles larvae and percentage reduction for different** ***Bti*** **concentrations (VectoBac® WDG) after larvicide application in open field trials from December 26th 2012 on**

	Average number per tub															Percentage reduction							
	Early instars					Late instars					Pupae					Early instars				Late instars			
Day	C	T1	T2	T3	T4	C	T1	T2	T3	T4	C	T1	T2	T3	T4	T1	T2	T3	T4	T1	T2	T3	T4
0*	35.0	35.0	35.0	35.0	35.0	35.0	35.0	35.0	35.0	35.0	0.0	0.0	0.0	0.0	0.0								
1	35.0	0.0	0.0	0.0	0.0	34.0	0.0	0.0	0.0	0.0	1.8	0.0	0.0	0.0	0.0	100	100	100	100	100	100	100	100
2	30.5	2.3	0.0	0.0	0.0	25.0	0.0	0.0	0.0	0.0	8.0	0.0	0.0	0.0	0.0	93	100	100	100	100	100	100	100
3	19.8	3.0	3.8	7.5	0.0	25.8	0.0	0.0	0.0	0.0	10.5	0.0	0.0	0.0	0.0	85	81	62	100	100	100	100	100
4	18.8	11.3	6.5	12.3	0.0	23.0	2.0	0.0	0.0	0.0	6.3	0.0	0.0	0.0	0.0	40	65	35	100	91	100	100	100
5	21.3	14.8	11.8	12.8	20.5	15.8	2.5	2.0	0.0	12.3	0.0	0.0	0.0	0.0	0.0	31	45	40	4	84	100	87	100
6	17.0	12.5	15.5	14.0	14.8	18.5	3.5	2.0	2.8	5.8	6.0	0.0	0.0	0.0	0.0	26	9	18	13	81	89	85	69
7*	9.5	7.3	14.8	9.8	10.3	17.3	10.3	4.5	5.8	8.8	8.5	0.0	0.0	0.0	0.0	24	0	0	0	41	74	67	49
8	6.3	0.5	0.0	0.0	0.0	15.0	0.0	0.0	0.0	0.0	5.8	0.0	0.0	0.0	0.0	90	100	100	100	100	100	100	100
9	7.3	0.5	0.0	0.0	0.0	9.5	0.0	0.0	0.0	0.0	3.8	0.0	0.0	0.0	0.0	91	100	100	100	100	100	100	100
10	5.0	0.5	0.0	0.0	0.0	7.8	0.0	0.0	0.0	0.0	1.8	0.0	0.0	0.0	0.0	87	100	100	100	100	100	100	100
11	18.5	19.0	0.0	7.8	17.3	6.5	0.0	0.0	0.0	0.0	3.0	0.0	0.0	0.0	0.0	0	100	59	14	100	100	100	100
12	20.8	17.0	4.0	13.0	19.3	6.5	1.8	0.0	0.0	1.0	4.3	0.0	0.0	0.0	0.0	0	88	39	14	55	100	100	70
13	17.8	11.3	6.5	12.3	13.3	6.3	7.5	0.0	1.0	4.5	1.8	0.0	0.0	0.0	0.0	17	76	33	31	0	100	52	0
14*	12.3	5.5	6.5	9.8	11.3	7.0	10.3	1.3	2.8	11.5	3.0	0.0	0.0	0.0	0.0	41	66	22	15	0	32	0	0
15	5.5	0.0	0.0	0.0	0.0	9.0	0.0	0.0	0.0	0.0	3.8	0.0	0.0	0.0	0.0	100	100	100	100	100	100	100	100
16	1.0	0.0	0.0	0.0	0.0	7.8	0.0	0.0	0.0	0.0	5.3	0.0	0.0	0.0	0.0	100	100	100	100	100	100	100	100
17	8.0	0.0	0.0	1.3	0.0	5.5	0.0	0.0	0.0	0.0	3.5	0.0	0.0	0.0	0.0	100	100	100	100	100	100	100	100
18	18.3	17.3	10.5	9.8	5.0	1.0	0.0	0.0	0.0	0.0	5.0	0.0	0.0	0.0	0.0	0	0	33	70	100	100	100	70
19	17.5	18.3	10.5	9.8	8.5	1.8	2.5	0.0	0.0	0.0	0.8	0.0	0.0	0.0	0.0	0	0	30	47	2	100	100	100
20	14.8	13.5	17.0	11.3	4.3	6.0	7.3	1.8	0.5	2.3	0.5	0.0	0.0	0.0	0.0	0	0	4	69	17	0	79	77
21*	8.0	8.5	16.5	10.5	2.3	8.3	12.3	2.5	1.5	3.3	3.3	0.0	0.0	0.0	0.0	0	0	0	69	0	0	54	76
22	7.0	0.0	0.0	0.0	0.0	4.8	0.8	0.0	0.0	0.0	3.0	1.0	0.0	0.8	0.0	100	100	100	100	89	100	100	100
23	11.0	0.0	0.0	0.0	0.0	6.8	0.8	0.0	0.0	0.0	0.8	0.0	0.0	0.0	0.0	100	100	100	100	100	100	100	100
24	12.0	0.0	0.0	4.0	0.0	4.8	0.8	0.0	0.0	0.0	3.0	0.0	0.0	0.0	0.0	100	100	75	100	89	100	100	100
25	14.8	8.0	0.0	6.0	0.0	4.5	0.0	0.0	0.0	0.0	1.3	0.8	0.0	0.0	0.0	49	100	69	100	100	100	100	100
26	13.5	14.8	0.0	9.8	5.0	5.0	0.0	0.0	1.0	0.0	1.3	0.0	0.0	0.0	0.0	0	100	45	0	100	100	0	100
27	13.0	10.0	2.0	11.5	4.8	6.3	3.8	0.0	1.0	0.3	3.3	0.0	0.0	0.0	0.0	28	93	33	0	60	100	12	90
28	12.0	8.3	5.8	10.3	2.8	3.8	8.5	1.3	2.5	2.3	3.3	0.8	0.8	0.0	0.0	35	77	35	19	0	0	0	0

*Asterisks indicate days with larvicide application. C = control, T_1_ = 0,2 mg/l, T_2_ = 0,4 mg/l, T_3_ = 0,8 mg/l, T_4_ = 1,0 mg/l Placeholder Table 2.

## Discussion

The residual effect varied between less than two days and up to three days, before new first instar larvae were found. The residual effect depends on the concentration of *Bti*. In both rounds the 0.2 mg/l concentration showed earlier larval reoccurrence than the others, notably during the first two applications. The fact that after the third and fourth application its efficacy was as good as those of the higher concentrations might have been induced by a slight drop in water levels in the test tubs. The second round of open field trials (from December 26th 2012 on) showed generally higher reduction rates in larval densities and slightly longer residual effects. Contributing factors might be the climatic situation in December, which shows lower air temperatures, decreased insolation and no diluting rainfalls. Increased insolation has been shown to lower the efficacy of *Bti*[16]; in Burkina Faso where the intensity of sunlight as well as the water temperature is high the potency of *Bti* formulations can decrease substantially. The lethal concentrations (L95) were much higher than under laboratory conditions. This is likely due to interactions with mud, vegetation, disturbance by animals and humans, high temperatures, insolation and relocation of surface water by strong winds.

The average water temperature during the first round was 27.1°C, whilst during the second round it was 25.0°C. Besides the *Bti* concentration, environmental conditions seem to have an influence on the larvicide’ s residual effect [17]. The precipitations occurring during the first round of tests do not seem to have affected the number of captured larvae. Relative air humidity [18] and the number of existing environmental breeding sites [19] have an influence on adult vector survival but did not result in differences in oviposition and hence container recolonization.

The current literature reports highly different findings on the residual effect. Kinde-Gazard & Baglo [20] reported a period of 9 days before larvae reappeared with a density of 1.4%. Kroeger *et al.*[21] found in a study carried out in Ecuador and Peru that an effective reduction of larvae was observed up to 7 to 10 days. For Eritrea Shililu *et al.*[22] described an effect up to two to three weeks. Majambere *et al*. [23] found first larvae in habitats 4 days after *Bti* treatment in the Gambia. Our findings are in line with other studies [4, 24, 25], which found an effect that lasted between two and three days and might be ascribed to a similar experimental setup. Difficulties in comparability of studies arise from different protocols and definitions of thresholds of effective larval reductions. Generally it can be distinguished between different study setups, e.g. trials under field, semi-field and laboratory conditions. Discrepancies between studies regarding the residual effect and efficacy may have their origin in the presence or absence of environmental parameters such as vegetation, insolation, and dilution. Furthermore, the definition of larvicidal activity seems inconsistent in literature; sometimes the effect on emerging adult mosquitoes or the reconolization with larvae is researched while other studies see the effect as the larvicide’s capability to still kill larvae.

Reapplication with the larvicide solution took place in weekly intervals. Fluctuations in larval densities may have their origin to some percentage in the larval dipping procedure but the biggest share is contributable to the development from one larval stage to another and the oviposition and development of new larvae. While larvae reoccurred within a relatively short time, virtually no pupae were able to develop between two *Bti* applications. Within the weekly intervention intervals late instar larvae were not capable of developing into pupae and imagines. Despite the short residual effect of all WDG Vectobac® *Bti* concentrations of approximately two days and the fast recolonization with larvae of all genera, the lack of pupae is significant and can be seen as the most important indicator for the efficacy of larviciding interventions [25]. We would suggest carrying out further studies to examine the possibility of decreasing the reapplication rates to longer intervals. Our observations of very low numbers of pupae towards the end of the treatment intervals might be an indicator for a prolonged protective effect of new adult mosquito emergence. Some studies indicate that after longer periods of continuous larvicide application the reconolization with larvae decreases. Nonetheless, further testing for the local appropriateness of application and persistence of other *Bti* formulations (e.g. VectoMax®) should be undertaken. Due to the conservation of natural aquatic predators attributable to the selective mode of action of *Bti* a permanent reduction of newly emerging larvae can be achieved and might result in a permanent residual control effect. Extensively used in many parts of the world, biological larviciding is to date sparsely implemented in African malaria control programs.

## Conclusions

The study shows that the WDG *Bti* formulation even at a very low dosage of 0.2 kg/ha is highly effective against the main malaria vector larvae in Burkina Faso, offering viable possibilities for larviciding in climatic and environmental conditions of tropical Africa. However, the observed short persistence might require frequent retreatment of breeding sites. *Bti* is a promising complimentary tool for integrated malaria control strategies for specific settings and future formulations with enhanced activity and persistence may extend its area of deployment as well as its cost effectiveness.
